# Postoperative tetraplegia due to conversion disorder upon emergence from general anesthesia

**DOI:** 10.1186/s40981-020-00394-9

**Published:** 2020-10-30

**Authors:** Asuka Ito, Tatsuo Nakamoto, Sayaka Ohira, Takahiko Kamibayashi

**Affiliations:** Department of Anesthesiology, Kansai Medical University, 2-3-1, Shin-machi, Hirakata, Osaka, 573-1191 Japan

**Keywords:** Conversion disorder, Tetraplegia, General anesthesia

## Abstract

**Background:**

Acute neurological deficit upon emergence from general anesthesia is a serious emergency. Conversion disorder, previously known as hysteria, is a somatoform disorder that causes neurological deficits without anatomical or physiological explanations. It is particularly rare after general anesthesia.

**Case presentation:**

A 28-year-old healthy Japanese woman presented tetraplegia with normal sensory function upon waking from general anesthesia. She was evaluated for the causes of tetraplegia. There were no abnormal findings, and her symptoms were inconsistent with any anatomical or neurological pathology. Although she could not flex her knee actively, she could maintain the passive flexed position, suggesting that her paralysis was nonorganic. The most likely diagnosis was conversion disorder. After a 12-h observation, the patient fully recovered.

**Conclusions:**

In patients with neurological deficits not correlating with neurological findings after general anesthesia, the presence of somatic disorders, such as conversion disorder, should be considered.

## Background

Postoperative neurological deficits, such as tetraplegia, are difficult to diagnose and manage by anesthesiologists. Acute neurological deficits after general anesthesia include a wide differential diagnosis. When confronted with less common and unexplained patterns of paralysis, clinicians are often obliged to perform diagnostic examinations to rule out organic causes, ranging from innocuous to life-threatening conditions. Therefore, immediate assessment and investigations are required.

Conversion disorder, previously known as hysteria, is defined as an altered voluntary motor or sensory function with clinical findings incompatible with recognized medical conditions [1]. Its incidence has been reported to be between 11 and 300 per 100,000 people in the general population [2]. Postoperative presentations of conversion disorder have rarely been reported and most patients had symptoms of hemiplegia [3] or paraplegia [4].

Here, we present a case of postoperative conversion disorder with tetraplegia that developed upon emergence from anesthesia. To our knowledge, this is the first report of a conversion disorder manifesting as symptoms of tetraplegia after general anesthesia.

## Case presentation

The patient was a 28-year-old Japanese woman, weighing 52 kg, with a right benign breast tumor. She had no history of neurological or psychiatric disease. The preoperative assessment was unremarkable. There seemed to be no history of neglect in her childhood or familial problems. She graduated from high school and was working for a company without any problems. The scheduled operation of her benign breast tumor was postponed for almost 2 months because of the COVID-19 pandemic. Therefore, she seemed to be very nervous and anxious about her disease. She underwent an excision of the breast tumor under general anesthesia. General anesthesia was induced with 150 mg of propofol, 100 mcg of fentanyl, and 50 mg of rocuronium. A #3 laryngeal mask airway (LMA) was inserted in the first attempt. Sevoflurane and remifentanil were used to maintain general anesthesia. Hemodynamic stability was maintained with only one dose of 4 mg of ephedrine. No local anesthetic agent was used during the procedure. Other intravenous medications administered intraoperatively were 100 mg of cefazolin and 6 mg of dexamethasone. For analgesia, 1000 mg of acetaminophen was administered. After the 1-h procedure, 200 mg of sugammadex was administered as a reversal agent after the recovery of spontaneous breathing. She demonstrated almost 450 mL of tidal volume, 10 times the respiratory rate per minute, and 0.2% of the end-tidal concentration of sevoflurane before the removal of the LMA.

In the operating room, 5 min after removal of the LMA, she was able to open her eyes voluntarily and open her mouth and put her tongue out when we indicated. She was alert and oriented, with normal vital signs. However, complete paralysis of bilateral lower extremities and incomplete paralysis of bilateral upper extremities were noted. Additionally, 200 mg sugammadex was administered. The train-of-four (TOF) ratio was 1.0 in the posterior tibial nerve and 0.92 in the ulnar nerve 10 min after the additional administration of sugammadex. She began to move her upper extremity but not the lower extremity. Laboratory tests, including the blood chemistry test, full blood count, electrolytes, and blood gas, were unremarkable.

Urgent magnetic resonance imaging (MRI) of the brain and spine was performed to exclude intracranial and spinal complications. It showed no structural abnormalities that would require emergent treatment. The neurology team was immediately consulted for further evaluation. A neurologist conducted neurological examinations 2 h after the removal of the LMA. The patient could maintain bilateral knee positions with passive flexion of her knee by the neurologist, while she could not flex her knee actively. Loss of sensation was not observed in any lesions. There was no hyperreflexia or hypotonia. These immediate results of her physical examination were inconsistent with the MRI findings. Ultimately, she was suspected to have a conversion disorder after other neurologic diseases were excluded. After a 12-h observation, she fully recovered and she could walk without any assistance and was discharged. At the 10-day follow-up, she demonstrated no neurological deficits.

## Discussion

The diagnosis of conversion disorder requires unexplained physical or sensory deficits involving the musculature and sensory system [1]. Symptoms of conversion disorder include the loss of physical function, such as amnesia, aphonia, pain, sensory deficits, blindness, and new-onset paralysis.

Our patient demonstrated tetraplegia upon emergence from general anesthesia and her consciousness was clear. Acute tetraplegia after general anesthesia is a serious emergency. The differential diagnosis for postoperative acute neurological deficit includes residual postoperative neuromuscular blockade, stroke, hematoma, seizures, intracranial infections, brain tumors, and metabolic disturbances. The possibility of residual paralysis was low because of the normal TOF ratio. Electrolytes were within normal limits. MRI showed no intracranial or spine lesions. A non-anatomic distribution of the deficit suggested a non-organic cause.

A neurologist conducted a neurological examination after we ruled out organic causes. Neurologists used the Spinal Injuries Center Test, developed in a prospective study by Yugue et al. [5]. This clinical evaluation test involves asking the patient to actively flex the knee and then passively flex the knee. The test is positive if the patient cannot actively flex the knee but maintains the passively imposed flex position. The sensitivity of this test can be improved by talking to distract the patient. It was positive in all patients with conversion paralysis in the literature [5].

We used the Spinal Injuries Center Test to differentiate conversion disorders. Another test to assist in distinguishing nonorganic from organic weakness is the Hoover test [6]. This sign relies on the principle of synergetic contraction. Involuntary extension of the pseudo-paralyzed leg occurs when flexing the contralateral, nonparalyzed leg against resistance. If downward pressure is felt from the contralateral heel while raising the nonparalyzed extremity, the weakness is likely nonorganic or psychiatric in nature. If no pressure is felt, the patient is likely to suffer from organic limb weakness.

Inorganic causes, such as somatic symptom disorders, should be considered when organic causes are ruled out. Somatic symptom disorders are physical symptoms that do not match any medical pathology. Conversion disorder is a type of somatoform disorder described in the Diagnostic and Statistical Manual of Mental Disorders, Fifth Edition (DSA-5) [1]. Conversion symptoms typically do not conform to known anatomical pathways or physiological mechanisms. They manifest as a physiological neurological problem unexplainable by neuroanatomy. This disorder is often seen in young women of lower socioeconomic status [1] and associated with family stress, grief, and adjustment difficulties [2].

The differential diagnosis of conversion disorder requires exclusion of other neurologic diseases and deliberate feigning. The DSA-5 requires the documentation of prior stressors associated with the development of conversion disorders, which are considered to be an unconscious psychological response to stressful situations. She seemed anxious and stressed regarding her disease and operation. Additionally, in patients with conversion disorder, a high rate of psychiatric comorbidity has been identified. Most patients have generalized anxiety disorder, dysthymic disorder, simple phobia, obsessive compulsive disorder, and major depression [7, 8]. We did not examine her psychiatric status preoperatively. A preoperative psychological assessment may help identify high risk patients for conversion disorder. Our patient fully recovered 1 day after the operation, and no neurological deficits were found subsequently. Therefore, we did not consult any psychologist. Ideally, examination and follow-up with a psychiatrist or psychologist might be recommended.

## Conclusions

We describe a case of tetraplegia upon emergence from general anesthesia. Once organic causes for neurological deficits are ruled out, conversion disorders should be considered in cases with unexplainable physical symptoms. In our case, the Spinal Injuries Center Test helped to distinguish non-organic weakness, such as conversion disorder, from organic weakness.

## Data Availability

Not applicable

## Abbreviations

LMA: Laryngeal mask airway
TOF: Train-of-four
MRI: Magnetic resonance imaging
DSA-5: Diagnostic and Statistical Manual of Mental Disorders, Fifth Edition
